# Addisonian Crisis in a 39-Year-Old Woman With Primary Adrenal Insufficiency

**DOI:** 10.7759/cureus.103607

**Published:** 2026-02-14

**Authors:** Mathew R Itteera, Maria Gutierrez, Krupal H Patel

**Affiliations:** 1 Family Medicine, Guthrie Lourdes Hospital, Binghamton, USA; 2 Hospital Medicine, Guthrie Lourdes Hospital, Binghamton, USA

**Keywords:** addisonian crisis, addison's disease, hypotension not responding to fluid resuscitation, hypotension workup, hypotensive emergency

## Abstract

Primary adrenal insufficiency, or Addison’s disease, is a rare endocrine disorder characterized by inadequate cortisol and aldosterone production. Addisonian crisis is a life-threatening complication triggered by physiological stress, infection, or medication non-adherence. We present the case of a 39-year-old Caucasian woman with a history of Addison’s disease, Hashimoto’s thyroiditis, celiac disease, vitiligo, and alopecia areata who presented with hypotension, bradycardia, and nausea secondary to an Addisonian crisis, likely precipitated by cyclic nausea and vomiting due to viral gastroenteritis, leading to an inability to tolerate oral medication. She was managed with intravenous fluids, pressors, and stress-dose steroids in the intensive care unit (ICU), with subsequent stabilization and transition to oral therapy. This case highlights the importance of early recognition, aggressive management, and patient education to prevent recurrent adrenal crises.

## Introduction

Primary adrenal insufficiency, or Addison’s disease, results from destruction or dysfunction of the adrenal cortex, leading to deficient production of cortisol and aldosterone. It has an estimated prevalence of 100-140 cases per million in Western populations [1]. Common causes include autoimmune adrenalitis, infections, and genetic disorders [2]. Addisonian crisis is an acute, life-threatening manifestation of adrenal insufficiency characterized by hypotension, electrolyte abnormalities, and altered mental status. It is often precipitated by stressors such as infection, trauma, or non-adherence to medications [3,4]. In addition to hypotension, other hallmark features may include hypoglycemia, tachycardia, abdominal pain, nausea, and vomiting. Typical electrolyte abnormalities include hyperkalemia and hyponatremia.

However, atypical presentations can occur in clinical practice. What makes this case unique is the absence of several classic features of Addisonian crisis, along with overlapping symptoms related to presumed viral gastroenteritis. If this presentation had occurred in a patient not previously established within a specific healthcare system, it could have led to diagnostic uncertainty. This case highlights the importance of maintaining a broad differential diagnosis to ensure timely recognition and management, including prompt administration of corticosteroids [4,5].

This report describes a 39-year-old woman with known Addison’s disease who presented with an Addisonian crisis likely triggered by persistent nausea and vomiting from gastroenteritis, resulting in the inability to tolerate oral medications. We discuss the clinical presentation, diagnostic evaluation, management, and strategies to prevent recurrence, emphasizing the critical role of early intervention.

## Case presentation

A 39-year-old Caucasian woman with a past medical history of Addison’s disease, Hashimoto’s thyroiditis, celiac disease, vitiligo, and alopecia presented to the emergency department (ED) with diaphoresis, headache, nausea, vomiting, and an inability to tolerate oral medications, including hydrocortisone, fludrocortisone, and levothyroxine.

On examination, the patient appeared acutely ill and somnolent. Vital signs revealed hypotension (blood pressure 75/48 mmHg), bradycardia (heart rate 46 beats per minute), and a temperature of 36.5°C. Physical examination was notable for cool, clammy skin and bronzed facial features consistent with chronic adrenal insufficiency. No focal neurological deficits were identified. The remainder of the examination was unremarkable.

**Figure 1:**
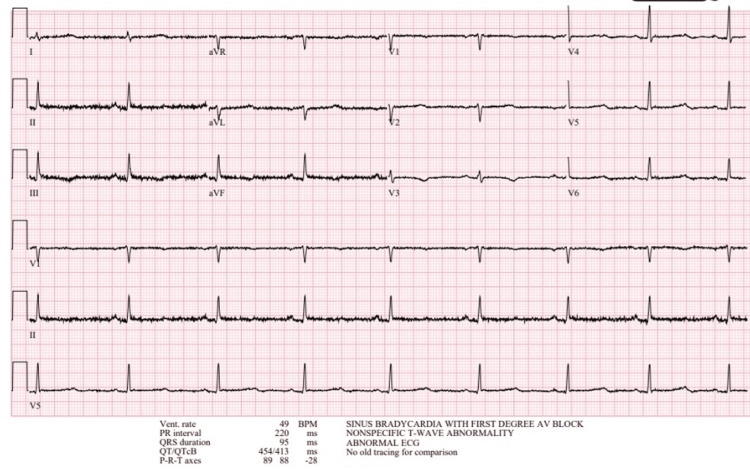
EKG showing sinus bradycardia and first-degree atrioventricular block

**Figure 2:**
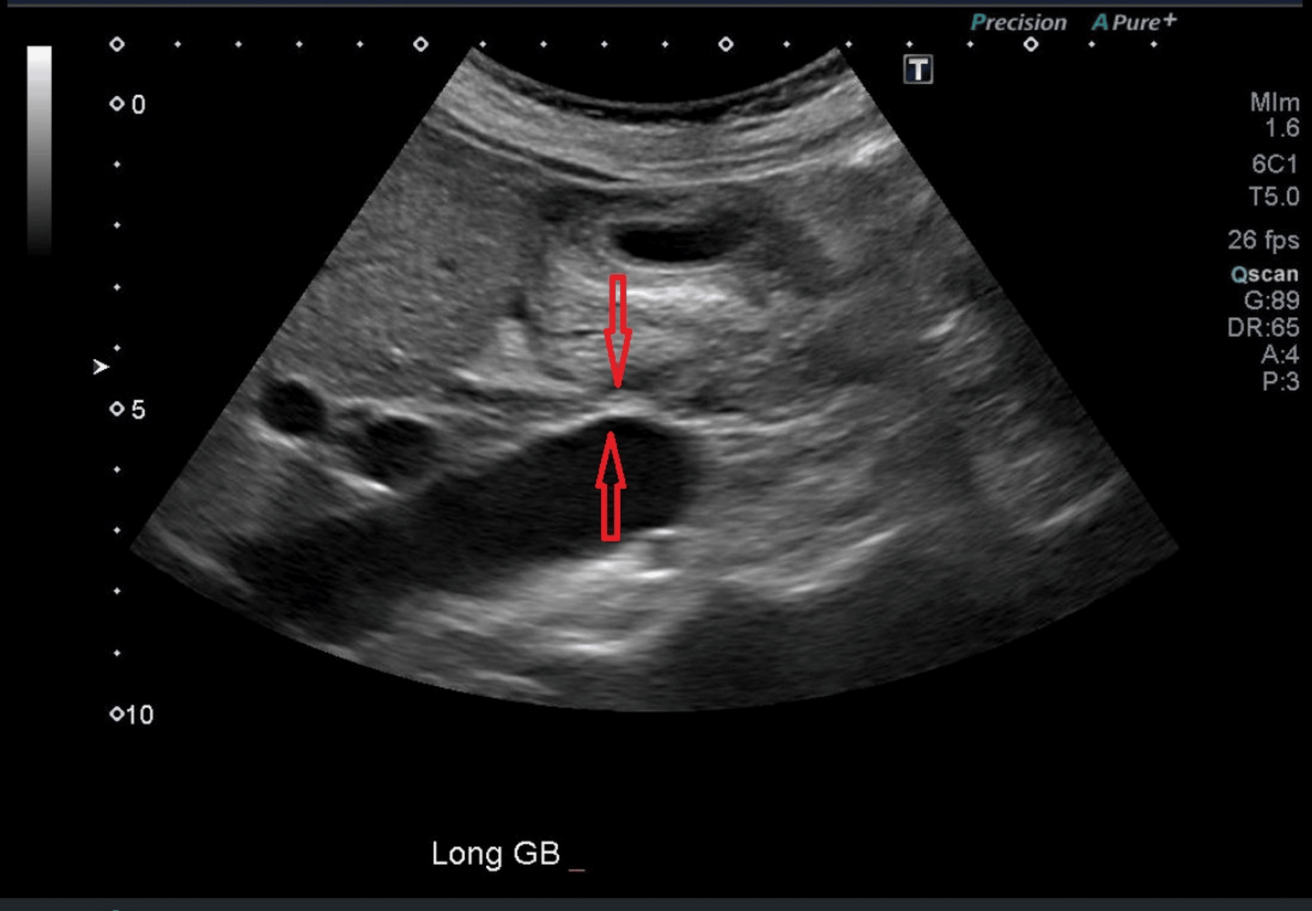
Abdominal ultrasound showing gallbladder wall thickening

In the ED, laboratory evaluation demonstrated hyponatremia (sodium 125 mmol/L; reference range: 135-145 mmol/L), normal serum potassium (3.9 mmol/L; reference range: 3.5-5.0 mmol/L), and normal blood glucose (71 mg/dL; reference range: 70-99 mg/dL). Liver enzymes were mildly elevated (aspartate aminotransferase 51 U/L and alanine aminotransferase 47 U/L; reference range: 10-35 U/L). Complete blood count was within normal limits. Lactate was not obtained during this admission. Renal function was normal, with an estimated glomerular filtration rate (eGFR) of 82 mL/min and a creatinine level of 0.91 mg/dL. Electrocardiogram demonstrated sinus bradycardia with first-degree atrioventricular block (Figure 1). Urinalysis was nonspecific, and urine culture showed no growth. Blood cultures and a respiratory viral panel were negative. Abdominal ultrasound revealed mild gallbladder wall thickening without evidence of cholelithiasis or cholecystitis (Figure 2). A stool panel was not obtained.

The patient received a 3 L bolus of normal saline, followed by maintenance intravenous fluids at 100 mL/hour, hydrocortisone 100 mg IV, fludrocortisone 0.1 mg orally, and norepinephrine for hemodynamic support. Broad-spectrum antibiotics (piperacillin-tazobactam 4.5 g intravenously every 8 hours) were initiated empirically to cover possible infectious triggers. She was admitted to the ICU for close monitoring.

In the ICU, the patient received 25 g of intravenous albumin and continued hydrocortisone 50 mg intravenously every 6 hours. Norepinephrine was gradually weaned as her blood pressure stabilized. After 48 hours, she was transitioned to oral hydrocortisone 30 mg daily and transferred to the medical floor. At the bedside, she reported recent exposure to a nephew with similar gastrointestinal symptoms, suggesting a viral etiology. Her hospital course was uncomplicated, with resolution of nausea and vomiting. She tolerated oral intake by hospital day 3 and was discharged on her home regimen of hydrocortisone, fludrocortisone, and levothyroxine. At discharge, her blood pressure had improved to 100/61 mmHg, and laboratory results showed a serum sodium level of 137 mmol/L.

At discharge, the patient was counseled on stress-dose steroid use, including administration of 100 mg intramuscular hydrocortisone from her emergency kit. The importance of medication adherence was reinforced. She was also prescribed antiemetics (ondansetron 0.4 mg every 4-6 hours as needed and metoclopramide 5 mg every 6-8 hours as needed) to manage nausea and prevent medication intolerance. She was advised that persistent nausea and vomiting significantly increase the risk of adrenal crisis and was instructed to return to the emergency department if symptoms prevented oral medication intake. Follow-up with her primary care provider and endocrinologist was arranged within one week, and she was discharged with an emergency hydrocortisone kit. 

## Discussion

Addisonian crisis is a medical emergency with significant mortality if untreated, resulting from acute deficiency of cortisol and aldosterone [2,4]. Common precipitants include infections, surgery, or inadequate glucocorticoid replacement during periods of stress [3,6]. In this case, the patient’s presentation with hypotension and hyponatremia was consistent with adrenal crisis, likely triggered by viral gastroenteritis, causing persistent nausea and vomiting with intolerance to oral medications.

The pathophysiology of Addisonian crisis involves an impaired stress response due to cortisol deficiency, leading to vasodilation, hypotension, and hypovolemia. This is compounded by aldosterone deficiency, which causes sodium loss and may result in hyperkalemia [2]. Bradycardia, as observed in this patient, is less commonly reported and may be related to electrolyte disturbances or autonomic dysregulation [7]. This differs from the more typical presentation of adrenal crisis, in which tachycardia is more frequently observed. According to Robbins and Cotran Pathologic Basis of Disease, autoimmune adrenalitis (likely in this patient, given her history of Hashimoto’s thyroiditis, vitiligo, and alopecia) is the leading cause of Addison’s disease in developed countries [2].

Management of Addisonian crisis, as outlined in the UCSF handbook, includes immediate administration of intravenous hydrocortisone (100 mg bolus followed by 50 mg every 6 hours), aggressive fluid resuscitation, and correction of electrolyte abnormalities [3-5]. Fludrocortisone is typically resumed for mineralocorticoid replacement once oral intake is tolerated. This patient’s rapid improvement following steroid therapy and fluid resuscitation underscores the effectiveness of guideline-directed management. Her reported exposure to a family member with gastroenteritis supports a stress-induced trigger in the setting of inability to tolerate oral medications. This aligns with UCSF recommendations emphasizing identification and management of precipitating causes [8].

Patient education is essential to prevent recurrence of adrenal crisis. Preventive strategies include stress-dose steroid protocols during illness, early use of antiemetics, and wearing medical alert identification [3,9]. This patient received counseling regarding the high risk posed by vomiting and diarrhea due to reduced absorption of hydrocortisone and increased cortisol requirements during illness. She was instructed on the use of a 100 mg intramuscular hydrocortisone rescue injection and advised to double or triple her usual oral steroid dose during febrile illness for the duration of symptoms. She was also strongly encouraged to wear a medical alert bracelet indicating her diagnosis of Addison’s disease [9,10].

A unique aspect of this case was the overlap of symptoms with viral gastroenteritis and the absence of several hallmark findings of adrenal crisis. The patient presented with nausea, vomiting, hypotension, and hyponatremia and reported a sick contact at home. However, she did not exhibit hypoglycemia, hyperkalemia, or tachycardia. Abdominal ultrasound demonstrated only mild gallbladder wall thickening, and the electrocardiogram showed bradycardia.

The nonspecific and overlapping symptoms of Addisonian crisis, such as nausea, vomiting, hypotension, and hyponatremia, can closely resemble more common conditions, including sepsis, acute abdominal pathology (e.g., gastroenteritis, appendicitis, or pancreatitis), and distributive or hypovolemic shock from other causes. In sepsis, patients often present with fever, leukocytosis, elevated inflammatory markers (e.g., procalcitonin or lactate), and an identifiable infectious source. In contrast, adrenal crisis more typically presents with persistent hypotension despite fluid resuscitation and vasopressor support, unexplained hyponatremia, and absence of marked leukocytosis or fever. Failure to consider adrenal insufficiency early may result in unnecessary imaging or surgical evaluation.

Risk factors for misdiagnosis include lack of a known history of adrenal insufficiency, overlapping gastrointestinal symptoms (as in this case), absence of classic laboratory findings such as hyperkalemia or hypoglycemia, and initial attribution of symptoms to more common conditions such as sepsis or dehydration. Delayed recognition is not uncommon, with up to 44% of patients diagnosed with adrenal insufficiency only after presenting in crisis. Clinicians should maintain a high index of suspicion in patients with refractory hypotension, unexplained hyponatremia, or a history suggestive of autoimmune disease. Early empiric administration of intravenous hydrocortisone (100 mg bolus) while awaiting confirmatory testing is appropriate, as timely corticosteroid replacement is lifesaving and may prevent progression to multiorgan failure [4,7].

If this patient had presented to a healthcare system without prior medical records or an established history, clinicians would need to maintain a broad differential diagnosis to ensure timely treatment. In cases of hypotension that do not respond to fluids and vasopressors, serum cortisol should be obtained, and high-dose corticosteroids should be administered without delay when adrenal crisis is suspected [4,7]. 

Limitations

Because the patient had an established diagnosis of Addison’s disease and responded promptly to treatment, abdominal CT imaging and cortisol level measurement were not performed. These studies may have provided additional diagnostic information regarding the etiology of her hypotension and hyponatremia.

## Conclusions

This case illustrates a presentation of Addisonian crisis in a patient with primary adrenal insufficiency, precipitated by gastroenteritis-associated nausea and vomiting that resulted in intolerance to oral medications. Prompt recognition, aggressive management with stress-dose steroids, intravenous fluids, and vasopressor support, along with comprehensive patient education, were key to a successful outcome. Clinicians should maintain a high index of suspicion for adrenal crisis in patients with Addison’s disease who present with hypotension and nonspecific symptoms to ensure timely intervention and prevent morbidity and mortality.

As discussed, adrenal crisis may present with overlapping symptoms and the absence of classic hallmark findings. Physicians must maintain a broad differential diagnosis when evaluating hypotension that does not respond to fluids and vasopressor support. In such cases, obtaining a serum cortisol level and initiating high-dose corticosteroids without delay is essential, as early treatment may be lifesaving.
